# Comment on Skjerve et al. Using Simulations to Help Public Health Students Overcome Language Barriers for Better Health Outcomes. *Int. J. Environ. Res. Public Health* 2023, *20*, 6259

**DOI:** 10.3390/ijerph23060781

**Published:** 2026-06-11

**Authors:** Rochele Villafuerte, Aaron Funa

**Affiliations:** 1 College of Education, Central Bicol State University of Agriculture, Calabanga Camarines Sur 4405, Philippines; 2 Graduate School, Sorsogon State University, Sorsogon City 4700, Philippines; funa.aaron@sorsu.edu.ph; 3 College of Health and Science, Sorsogon State University, Sorsogon City 4700, Philippines

Skjerve et al. [1] address an important issue in contemporary public health education. As health systems become increasingly multilingual and culturally diverse, language barriers remain a persistent obstacle to effective communication, equitable care, and meaningful health engagement [2,3]. In this context, the authors offer a valuable educational perspective by examining how simulation can prepare public health students for challenging encounters with clients who do not share a common language. For example, the facilitators observed that several groups forgot to ask about the client’s mother tongue and instead defaulted to asking whether the client could communicate in English, revealing how easily students may overlook basic but essential communication assessment steps in multilingual encounters [1]. A notable strength of the paper is its emphasis on repeated simulation and debriefing, both of which are widely recognized as important features of effective simulation-based learning [4,5]. More recent literature further supports the value of simulation in culturally and linguistically diverse health education contexts, particularly for developing communication confidence, health literacy support, and inclusive learning experiences in language-discordant encounters [6,7].

From a public health perspective, the value of simulation extends beyond the development of individual communication skills. Language barriers may restrict access to care, weaken health literacy, and deepen inequities among migrants, ethnic minorities, older adults, and other populations who may have limited proficiency in the dominant language of care [2,3,8]. Simulation-based training can therefore be understood as an equity-oriented pedagogical strategy because it allows students to practice identifying language needs, working with interpreters, checking understanding, and adapting messages to the client’s cultural and literacy context. In this way, language-responsive simulation supports not only safer provider–patient communication but also more inclusive public health engagement.

At the same time, several considerations may help contextualize the findings. First, the study may be understood as an exploratory and context-specific educational investigation, which provides a useful basis for future studies in other settings. The sample was small and drawn from a single institution, which may limit transferability to other educational settings. Second, the qualitative evidence was derived from facilitator logbooks, questionnaires, and evaluation forms rather than audio- or video-recorded interaction data. While this approach appropriately captures reflection and perceived learning, future studies could extend the design by analyzing recorded simulated encounters through discourse analysis, conversational analysis, or structured communication coding frameworks. These approaches may allow closer examination of how students manage turn-taking, repair misunderstandings, seek clarification, use interpreters, check comprehension, and adapt messages in culturally and linguistically complex encounters. This consideration is especially relevant because the study itself reports specific interactional difficulties, such as students repeating words the client did not understand without further explanation, which could be examined more closely through recorded interaction data [1]. Third, although the article demonstrates immediate educational value, it was not designed to determine whether the observed gains are sustained over time or transferred to authentic public health settings [1].

These considerations do not diminish the importance of the article. Rather, they identify promising directions for future work. Future studies could extend this exploratory mixed-methods design through more concrete research lines, such as using standardized patients from different linguistic backgrounds, incorporating interpreter-mediated scenarios, applying validated instruments to assess intercultural competence and communication performance, and adopting mixed longitudinal designs to examine whether learning gains are sustained over time and transferred to real-world public health settings. This would be particularly useful because the original study noted that all students performed better in the second interview after debriefing, suggesting that improvement occurred, but without longer-term evidence it remains unclear whether such gains would persist beyond the immediate simulation context [1]. These extensions would provide additional evidence on whether simulation-based training supports durable communication performance, culturally responsive practice, and equitable engagement in real-world public health environments [3,4,8].

Overall, the article offers a useful foundation for integrating language-responsive, culturally aware, and equity-oriented simulation into public health curricula. Its contribution is timely and relevant, and it can serve as a starting point for further investigations using complementary methods to examine how simulation may help future health professionals address language barriers more effectively.
